# Effect of Prophylactic Colon ESD (Endoscopic Submucosal Dissection) Defect Closure on Post-ESD Outcomes: An International Multi-center Retrospective Study

**DOI:** 10.1007/s10620-025-09518-6

**Published:** 2025-11-05

**Authors:** Rahul Karna, Jonathan Colón Sánchez, Kevan Josloff, Tammy Tran, Kasenee Tiankanon, Saowanee Ngamruengphong, Elisabet Maristany Bosch, Georgios Kalopitas, Edward John Despott, Alberto Murino, Shaimaa Elkholy, Mohamed El Sherbiny, Karim Essam, Hany Haggag, Abeer Awad Abdallatef, Kerolis Yousef, Rossella Maresca, Federico Barbaro, Galen Leung, Frances Dang, Amirali Tavangar, Jason Samarasena, Ahmed Saeed, Sherif Andrawes, Yutaka Tomizawa, Mohammad Bilal, Kartik Sampath, Yasi Xiao, Faisal Kamal, Thomas Kowalski, Alexander Schlachterman, Anand R. Kumar

**Affiliations:** 1 Division of Gastroenterology & Hepatology, University of Minnesota, Minneapolis, MN 55414 USA; 2 Department of Internal Medicine, Thomas Jefferson University Hospital, Philadelphia, PA USA; 3 Division of Gastroenterology & Hepatology, Department of Medicine, Thomas Jefferson University Hospital, Philadelphia, PA USA; 4 Division of Gastroenterology & Hepatology, Johns Hopkins Medicine, Baltimore, MD USA; 5 Division of Gastroenterology, Department of Medicine, Faculty of Medicine, Chulalongkorn University and King Chulalongkorn Memorial Hospital, Bangkok, Thailand; 6 Institute for Liver & Digestive Health, UCL (University College London), The Royal Free Hospital, London, UK; 7 Gastroenterology Division, Internal Medicine Department, Faculty of Medicine, Cairo University Kasr Alainy, Cairo, Egypt; 8 Digestive Endoscopy Unit, Fondazione Policlinico Universitario Agostino Gemelli IRCCS, and Università Cattolica del Sacro Cuore, Rome, Italy; 9 Division of Gastroenterology & Hepatology, New York University Langone Health, New York City, NY USA; 10 Division of Gastroenterology, University of California Irvine, Orange, CA USA; 11 Division of Gastroenterology, Research Medical Center, Kansas City, MO USA; 12 Division of Gastroenterology and Hepatology, Staten Island University Hospital-Northwell Health, Staten Island, NY USA; 13 Division of Gastroenterology, University of Washington, Seattle, WA USA; 14 Division of Gastroenterology & Hepatology, University of Colorado Anschutz Medical Campus, Aurora, CO USA; 15 Division of Gastroenterology, New York Presbyterial Hospital, Weill Cornell Medical Center, New York City, NY USA

**Keywords:** Defect closures, Delayed adverse events, Endoscopic submucosal dissection, Bleeding, Perforation, Colon

## Abstract

**Background and Aims:**

Prophylactic colonic endoscopic submucosal dissection (ESD) defect closure may reduce delayed adverse events (DAEs) such as bleeding and perforation associated with ESD and facilitate same day discharge. We compared the effect of colonic ESD defect closure (closed group) with no closure (open group) on DAEs and overnight hospital admission.

**Methods:**

We performed a Western multicenter retrospective study on patients who underwent colon ESD. Rectal lesions were excluded. DAEs were defined as adverse events within 2 weeks of ESD. Primary outcome measures were DAEs and overnight hospital admission. Multivariate analyses were performed.

**Results:**

560 patients underwent colon ESD and 364 (71.8%) patients had complete defect closure. Closed group had a significantly lower rate of delayed bleeding (1.7% vs 5.6%, *p* = 0.03) compared to open group. Multivariate analysis with adjusted odds ratios (aOR) revealed right sided polyps (aOR = 7.0) and anticoagulation/antiplatelet agents (aOR = 6.6) increased the risk while defect closure (aOR = 0.2) decreased the risk of delayed bleeding. Defect closure amplified the reduction in risk of delayed bleeding (2.4% vs 10.4%, *p* = 0.014) for right-sided polyps. Malignant polyps significantly increased the risk of delayed perforation (aOR = 3.3) and overnight hospitalization (aOR = 2.9). Defect closure (aOR = 0.6), traction use (aOR = 0.6) and topical hemostatic agent use (aOR = 0.4) significantly reduced the risk of overnight hospitalization.

**Conclusion:**

Prophylactic closure of colon ESD defects was associated with a significant reduction in delayed bleeding with number needed to treat (NNT) of 25.6 (especially for right sided polyps, NNT 12.5), and post-procedural overnight hospitalization. Prospective studies are needed to further validate these results.

**Supplementary Information:**

The online version contains supplementary material available at 10.1007/s10620-025-09518-6.

## Introduction

Endoscopic submucosal dissection (ESD) is increasingly utilized for resection of large non-pedunculated colorectal polyps (LNPCPs). In some countries, it has become the standard of care to achieve en-bloc specimen for accurate histopathological analysis and risk stratification for lymph node metastases. Previous meta-analyses have reported a higher *en-bloc r*esection rate of 90% with ESD compared to 50% with endoscopic mucosal resection (EMR) [1, 2]. A recent multicenter randomized controlled trial, assessing LNPCPs > 25 mm demonstrated significantly lower 6-month recurrence (0.6%) with ESD compared to 5.1% with EMR [3]. These outcomes favor the utilization of ESD over EMR for removal of LNPCPs. However, these advantages of ESD come at an increased risk of intraprocedural adverse events and delayed adverse events (DAE) due to the technical complexity of ESD in the colon [1–3]. Intraprocedural adverse events can be managed mostly endoscopically, however DAEs lead to repeat hospitalizations, blood transfusions and endoscopic/surgical procedures. Most commonly observed DAE within 2 weeks post ESD include bleeding and perforation, which are reported in up to 8% and 0.5% respectively [4, 5].

Despite the advantages of ESD, lack of resources, expertise and higher adverse event rate limit widespread adoption of ESD in the West. Thus, some endoscopists favor EMR for removal of LNPCPs. Recent randomized controlled trials have established benefit of prophylactic clipping after EMR in the colon to prevent post-procedure delayed bleeding, but not for delayed perforation [6, 7]. These benefits were amplified in the right colon (proximal to hepatic flexure). However, there are differences in resection technique (knife vs snare) and hemostasis technique (prophylactic coagulative hemostasis of individual vessels with knife or forceps vs blended coagulation over a large area with snare) between ESD and EMR. Hence, ESD defects may not have similar associated risks as EMR defects. In addition, our recent experience with defect closure after ESD in the rectum showed no significant reduction in DAE with non-significant trends of DAE reduction in high-risk groups. There was, however, significant reduction in the overnight hospital admission [8]. Currently, American Gastroenterological Association and European Society of Gastrointestinal Endoscopy do not favor routine closure of defects after colon ESD due to small retrospective studies or studies that have not separated colon from rectum [9–12].

Overall, there is inadequate evidence on the effect of prophylactic ESD defect closure in the colon. We conducted an international multicenter retrospective study in the West to evaluate the effect of prophylactic complete defect closure with multiple closure techniques on the rates of DAEs and overnight hospital admission after colonic ESD.

## Methods

We conducted an international multicenter retrospective study of patients who underwent ESD for colon polyps between 2017 and 2024. Data was collected across 11 centers (United States:8, United Kingdom:1, Italy:1 and Egypt:1). The indications for ESD, techniques and decision regarding defect closures or trainee involvement during procedure were at the discretion of the endoscopist. Peri-operative management of antithrombotic and antiplatelet agents was as per American Society for Gastroenterology or European Society of Gastrointestinal Endoscopy guidelines., as followed locally [13, 14]. Data was collected only on patients without intraprocedural perforations. This study was approved by the institutional review board at individual institutions.

### Data Extraction and Recording

Demographic details of patients including age, antiplatelet and anticoagulant use, history of prior manipulation of polyp site (such as EMR, snare polypectomy or surgery) were collected. Polyp characteristics including size and location (right sided vs left sided polyps) were recorded. All procedures were performed by endoscopists with at least two years of experience performing ESD. Procedural data was recorded, including traction use, topical hemostatic agents (Purastat, Nexpowder, Endoclot) and defect closure. When complete closure was performed, type of closure, such as use of suturing devices including over the scope suturing system (OTSS, Overstitch; Boston Scientific, Marlborough, Mass, USA), through the scope suturing system (TTSS, X-Tack Endoscopic HeliX Tacking System, Boston Scientific, Marlborough, Mass, USA), through-the-scope (TTS) clips including endoclips and tissue approximation clips like Mantis (Boston Scientific, Marlborough, Mass, USA) or Dual Action Tissue (DAT) clip (Micro-Tech Endoscopy, USA Inc, Ann Arbor, Michigan, USA), or combination of any of these techniques were recorded. Outcomes data including resection details (complete or incomplete), degree of submucosal fibrosis and procedure time, histopathology as having high grade dysplasia (HGD) or cancer, hospitalization and adverse events were also recorded.

### Study Outcomes

**Fig. 1:**
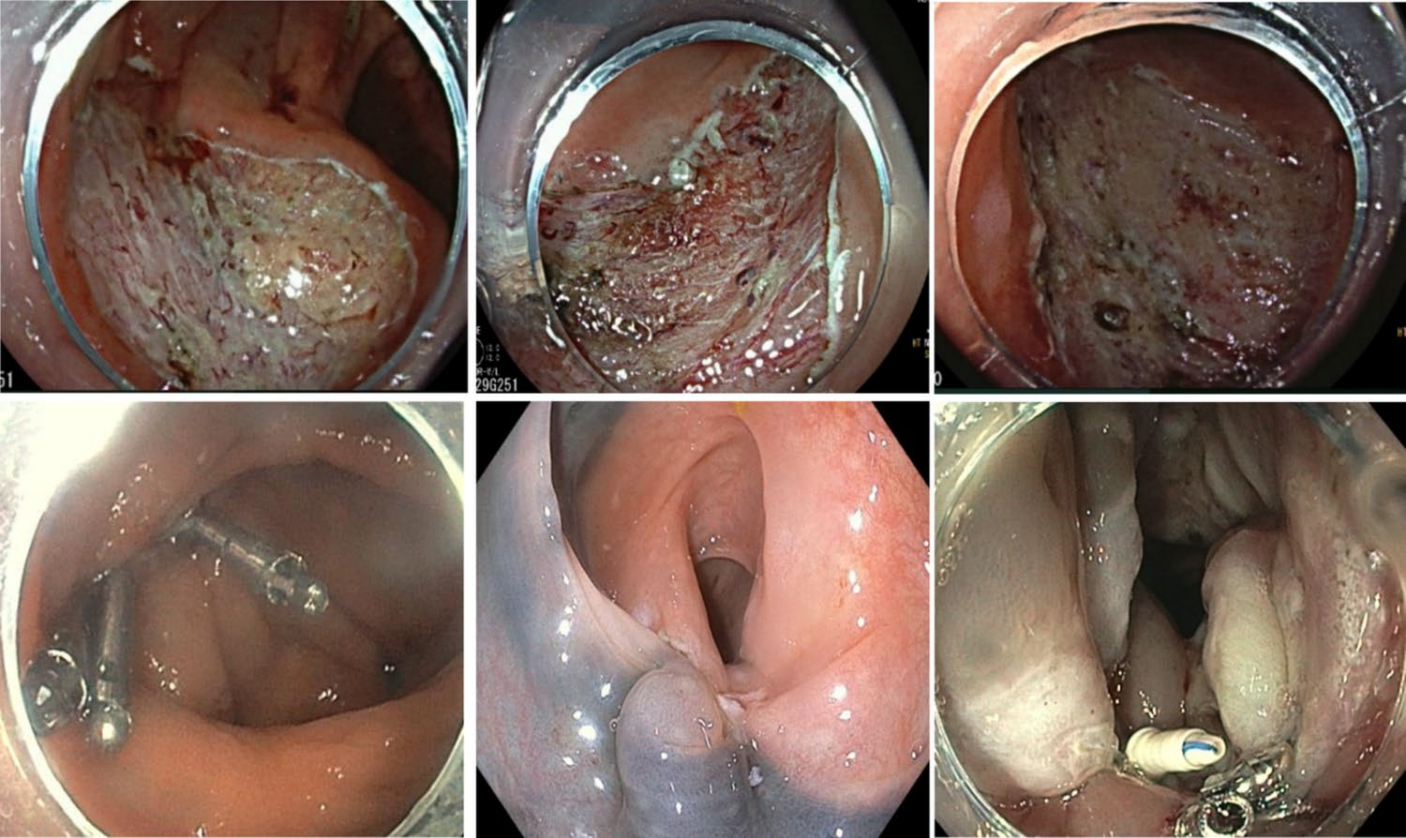
(Top row) Demonstration of open defects after colonic ESD; (bottom row from left to right) defect closure with through-the-scope clips (Resolution™), defect closure with over-the-scope suturing system (OverStitch™), defect closure with through-the-scope suturing system (X-Tack™)

The primary outcome of this study was to compare the post-colon ESD DAEs between open and closed groups. Secondary outcomes were predictors of delayed adverse events and impact of defect closure on post-procedural hospital admission for observation. Patients with completely closed ESD defects were placed in the “closed” group, while those without attempted closure were in the “open” group. Figure 1 demonstrates examples of closed and open defects after colonic ESD. Patients with partially closed defects were excluded from the outcomes analysis for defect closure.

### Study Definitions

1. Closed defects: Closed defects were defined as no exposed submucosa after defect closure by the performing endoscopist. The distinction between closed and open ESD defects was determined by the endoscopist performing the procedure at the end of the procedure.

2. Fibrosis: The degree of submucosal fibrosis was determined by the endoscopist as follows: F0, no fibrosis; F1, mild fibrosis; and F2, severe fibrosis characterized by a white, wall-like appearance [15].

3. Procedure time: The procedure time in our study was defined as time taken for completion of procedure and included time taken for defect closure in the “closed group”

4. Delayed adverse events: DAEs were defined as all adverse events that led to deviation from standard post-procedure course with need of an intervention (blood transfusion, antibiotics, endoscopy, surgery or unplanned hospitalization) within 2 weeks of ESD.

5. Delayed bleeding: Delayed bleeding was defined as hematochezia requiring endoscopy, surgery or unplanned hospital admission for blood transfusion or observation, within 2 weeks of ESD.

6. Delayed perforation: Delayed perforation was defined based on clinical and/or radiographic features that required intervention (endoscopy, surgery, or unplanned hospital observation) within 2 weeks of ESD.

Management of DAEs including hospital admission for observation, antibiotics, blood transfusion, repeat colonoscopy or surgery were recorded. All adverse events were graded as per AGREE classification [16]. Briefly, AGREE grade 1 adverse events do not result in deviation from normal post-procedure course (not captured in this study), grade 2 result in pharmacologic intervention, grade 3 require surgical, endoscopic or radiological intervention with (3a) or without (3b) general anesthesia, grade 4 require ICU admission and grade 5 are fatal adverse events.

### Statistical Analysis

All statistical analyses were performed using statistical software (SPSS v30.0.0.0). Categorical variables were compared in percentages and continuous variables as mean ± standard deviation. Comparison between categorical variables was evaluated using chi square test or Fisher’s exact test. Continuous variables were assessed using a t-test. Covariates with value of 0.10 or less were included in regression analysis in a stepwise method. Multivariate logistic regression analysis was performed to assess the risk factors for DAEs and overnight hospital admission. Subgroup analyses were performed where indicated. The *p*-value < 0.05 and odds ratios with 95% confidence intervals not crossing 1 were considered as measures of statistical significance.

## Results

### Baseline Details of Included Cohorts

**Fig. 2:**
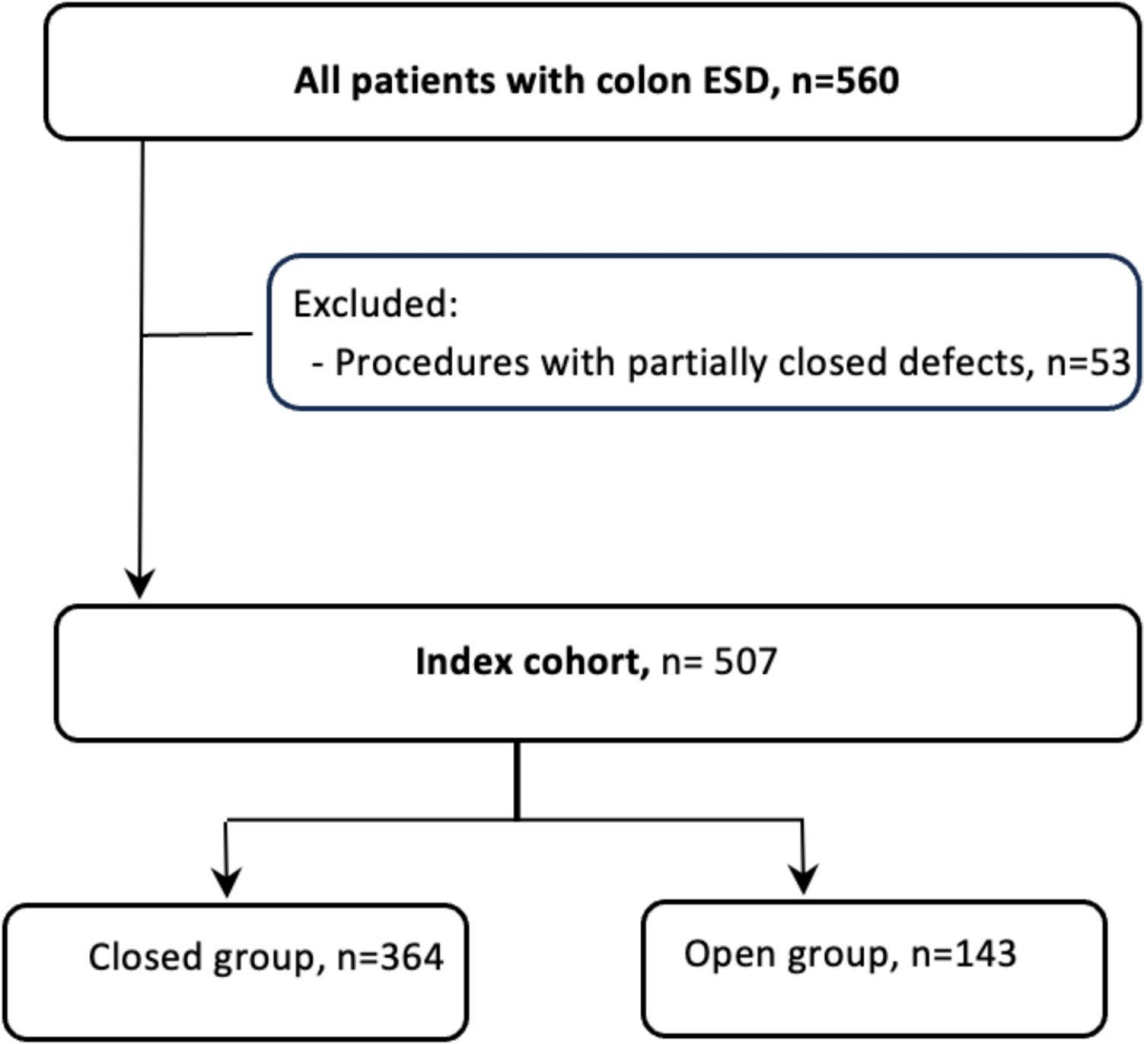
Flowsheet demonstrating development of the analytic cohort

The study included 560 patients with mean age of 64 ± 11.6 years who underwent colonic ESD without intraprocedural perforations between 2017 and 2024 across 12 centers. To assess the effects of defect closure on primary outcome measures, 53 patients with partial defect closure were excluded. Figure 2 demonstrates a flowsheet demonstrating patient selection. Complete defect closure was achieved in 364 (71.8%) patients. Distribution of age and other variables between closure and open groups are shown in (Tables 1 and 2). In the closure group, there were significantly higher Paris 0-Is or 0-Isp lesions (58.5% v 43%, *p* = 0.002), more high-grade dysplasia (30.5% v 14.7% *p* < 0.001), less malignant polyps (11.3% v 18.2%, *p* = 0.03), less incomplete resections (2.2% vs 7%, *p* = 0.009) and less topical hemostatic agent use (5.2% v 21%, *p* < 0.001) compared to the open group. There were no other significant differences in baseline characteristics between the closure and open groups.

**Table 1:** Baseline and polyp characteristics of included patients in closed and open ESD defects cohort

Total number of patients (*n* = 507)	Closure group (*n* = 364)	Open group (*n* = 143)	*p*-value
Age in years, mean (s.d.)	63.5 (12.2)	64.6 (10.5)	0.347
Antiplatelet or anticoagulation use, n (%)	41 (11.3%)	13 (9.1%)	0.475
History of previous intervention*, n (%)	76 (20.9%)	22 (15.4%)	0.159
Right sided polyps, n (%)	172 (47.3%)	67 (46.9%)	0.935
Paris 0-Is or 0-Isp, n (%)	203 (58.5%)	61 (43%)	**0.002**
Paris 0-IIb, n (%)	55 (16.2%)	32 (22.5%)	0.098

*ESD* endoscopic submucosal dissection, *n* number, *s.d.* standard deviation

^*^history of endoscopic mucosal resection, snare polypectomy or surgery

The p-value 0.05 and odds ratios with 95% confidence intervals notcrossing 1 were considered as measures of statistical significance

**Table 2:** Procedural details and outcomes included patients in closed and open ESD defects cohort

Total number of patients (*n* = 507)	Closure group (*n* = 364)	Open group (*n* = 143)	*p*-value
Presence of Fibrosis, n (%)	179 (49.2%)	75 (52.4%)	0.507
Prophylactic hemostasis n (%)	217 (59.6%)	81 (56.6%)	0.541
Topical hemostatic gel/powder, n (%)	19 (5.2%)	30 (21%)	** < 0.001**
Purastat	17 (4.7%)	25 (17.4%)	
Nexpowder	1 (0.3%)	0	
Endoclot	1 (0.3%)	4 (2.8%)	
Other	0	1 (0.7%)	
Traction use, n (%)	99 (27.2%)	33 (23.1%)	0.341
Incomplete resection, n (%)	8 (2.2%)	10 (7%)	**0.009**
Polyp size in mm, mean (s.d.)	42.7 (19.8)	43.9 (21.5)	0.535
High grade dysplasia, n (%)	111 (30.5%)	21 (14.7%)	** < 0.001**
Malignant Polyps, n (%)	41 (11.3%)	26 (18.2%)	**0.038**
Procedure time in minutes, mean (s.d.)	160.6 (125.4)	154.8 (111.1)	0.641
Type of closure device		NA	NA
Over the scope suturing system (Overstitch)	42 (11.8%)		
Through the scope suturing system (X-Tack)	68 (19.1%)		
TTS Clips	153 (43%)		
Mantis Clip	15 (4.2%)		
DAT Clip	1 (0.3%)		
Combination methods	77 (21.6%)		

*ESD* endoscopic submucosal dissection, *n* number, *S.d.* standard deviation, *TTS* through the scope, *DAT* dual action tissue

The* p*-value 0.05 and odds ratios with 95% confidence intervals not crossing 1 were considered as measures of statistical significance

### All Delayed Adverse Events (DAE)

Delayed adverse events were recorded in 9.1% (51/560) with the majority being bleeding, perforation and pain (Table 3). Severity of DAEs were recorded as grade II in 62.7% (32/51); grade IIIa in 19.6% (10/51) and grade IIIb in 17.6% (9/51) as per AGREE classification. There were no deaths associated with the procedure. On univariate analysis, large polyp size (51.7 mm vs. 42.2 mm, *p* < 0.01) and longer procedure time (242.1 min. vs. 148.5 min., *p* < 0.01) were significantly associated with higher DAEs (Table 4). On multivariate logistic regression analysis, only procedure time remained significantly (*p* = 0.003) associated with all DAEs. In patients with longer procedure times (> 180 min), there was a numerically lower rate of DAEs in the closed group compared to the open group with borderline statistical significance (13.5% vs 28.1%, *p* = 0.053).

**Table 3:** Delayed adverse events after endoscopic submucosal dissection and management

Type of adverse events	Management		
	Endoscopy	Surgery	Conservative
Delayed bleeding *n* = 15	5	1	9
Delayed perforation *n* = 21	4	8	9
Others*, *n* = 15	1		14

^*^Others included abdominal pain (*n* = 8), sepsis (*n* = 2), fever (*n* = 3), acute kidney injury (*n* = 1), dysphagia (*n* = 1)

The* p*-value 0.05 and odds ratios with 95% confidence intervals not crossing 1 were considered as measures of statistical significance

**Table 4:** Univariate and multivariate analysis for predictors of all delayed adverse events (DAE)

	DAE (*n* = 51)		*p*-value
Univariate analysis			
Antiplatelet/anticoagulant use, Yes vs No, n (%)	9 (15%)	42 (8.4%)	0.093
Prior intervention, Yes vs No, n (%)	14 (13.2%)	37 (8.1%)	0.103
Right sided polyps, Yes vs No, n (%)	29 (10.7%)	22 (7.6%)	0.204
Paris 1 s or 1sp, Yes vs No, n (%)	26 (8.8%)	21 (8.5%)	0.898
Paris 2b, Yes vs No, n (%)	8 (8.6%)	39 (8.8%)	0.945
Traction, Yes vs No, n (%)	9 (6%)	42 (10.2%)	0.129
Submucosal fibrosis, Yes vs No, n (%)	27 (9.3%)	24 (8.9%)	0.884
Incomplete vs Complete resection, n (%)	4 (19%)	47 (8.8%)	0.115
Prophylactic hemostasis, Yes vs No, n(%)	35 (10.8%)	16 (6.8%)	0.105
Closed vs Open defects, n (%)	33 (9.1%)	16 (11.2%)	0.467
Topical hemostatic agent, Yes vs No, n (%)	5 (7.4%)	46 (9.3%)	0.592
HGD, Yes vs No, n (%)	18 (12.8%)	33 (7.9%)	0.081
Malignant polyp, Yes vs No, n (%)	8 (10.8%)	43 (8.8%)	0.585
Mean (s.d.) age in years (DAE v no DAE)	65.2 (10.9)	63.9 (11.6)	0.432
Mean (s.d.) procedure time in min	242.1 (182.3)	148.5 (104.9)	** < 0.001**
Mean (s.d.) polyp size in mm	51.7 (26)	42.2 (19.3)	**0.001**
Multivariate analysis			
Procedure time (min)	0.996 (0.994–0.999)		**0.003**

*n* number, *s.d.* standard deviation, *HGD* high grade dysplasia, *DAE* delayed adverse events

The* p*-value 0.05 and odds ratios with 95% confidence intervals not crossing 1 were considered as measures of statistical significance

### Delayed Bleeding

Delayed bleeding was seen in 2.7% (15/560) patients in our cohort. Severity of delayed bleeding was recorded as grade II in 60% (9/15); grade IIIa in 33.3% (5/15) and grade IIIb in 6.7% (1/15) as per AGREE classification. Closed group had significantly lower rates of delayed bleeding compared to open group (1.7% vs 5.6%, p-value 0.03). Multivariate regression analysis revealed right sided polyps (aOR:7.0, *p* = 0.017) and anticoagulation/antiplatelet agents (aOR: 6.6, *p* = 0.007) increased the risk of delayed bleeding while defect closure (aOR: 0.2, *p* = 0.03) decreased the risk of delayed bleeding (Table 5). In patients with open defects, topical hemostatic agent use was associated with no delayed bleeding compared to 7.1% delayed bleeding when topical agent was not used. This difference was, however, not statistically significant (*p* = 0.2).

**Table 5:** Univariate and multivariate analysis for predictors of delayed bleeding

	Delayed bleeding (*n* = 15)		*p*-value
Univariate analysis			
Antiplatelet/anticoagulant use, Yes vs No, n (%)	4 (6.7%)	11 (2.2%)	0.068
Prior intervention, Yes vs No, n (%)	4 (3.8%)	11 (2.4%)	0.501
Right sided polyps, Yes vs No, n (%)	12 (4.5%)	3 (1%)	**0.013**
Paris 1 s or 1sp, Yes vs No, n (%)	8 (2.8%)	5 (2%)	0.586
Paris 2b, Yes vs No, n (%)	3 (3.3%)	10 (2.3%)	0.481
Traction, Yes vs No, n (%)	3 (2%)	12 (2.9%)	0.769
Submucosal fibrosis, Yes vs No, n (%)	7 (2.4%)	8 (3%)	0.686
Incomplete vs Complete resection, n (%)	4 (19%)	11 (2.1%)	** < 0.001**
Prophylactic hemostasis, Yes vs No, n (%)	12 (3.8%)	3 (1.3%)	0.074
Closed vs Open defects, n (%)	6 (1.7%)	8 (5.6%)	**0.030**
Topical hemostatic agent, Yes vs No, n (%)	0	15 (3.1%)	0.236
HGD, Yes vs No, n (%)	6 (4.3%)	9 (2.2%)	0.225
Malignant polyp, Yes vs No, n (%)	0	15 (3.1%)	0.240
Mean (s.d.) age in years (bleed vs no bleed)	66.6 (10.5)	63.9 (11.6)	0.389
Mean (s.d.) procedure time in min (bleed v no bleed)	285.9 (219.9)	152.93 (111)	** < 0.001**
Mean (s.d.) polyp size in mm (bleed vs no bleed)	46.6 (22)	43.1 (20.2)	0.525
Multivariate analysis			
Right sided polyps	7.0 (1.4–3.4)		**0.017**
Antiplatelet/anticoagulant use	6.6 (1.7–26.4)		**0.007**
ESD defect closure	0.2 (0.1–0.9)		**0.033**

*n* number, *s.d.* standard deviation, *HGD* high grade dysplasia, *DAE* delayed adverse events, *ESD* endoscopic submucosal dissection

The* p*-value 0.05 and odds ratios with 95% confidence intervals not crossing 1 were considered as measures of statistical significance

Subgroup analysis was performed for the two groups of patients with higher risk of delayed bleeding on multivariate analysis. In those with right-sided polyps, defect closure significantly reduced rates of delayed bleeding (2.4% vs 10.4%, *p* = 0.014). Rates of delayed bleeding did not differ significantly between closed and open groups in patients with anticoagulant/antiplatelet use (7.3% vs 7.1%, *p* = 1.0).

### Delayed Perforation

Delayed perforation was seen in 3.7% (21/560) patients in our cohort. Severity of delayed perforation was recorded as grade II in 42.8% (9/21); grade IIIa in 19.1% (4/21) and grade IIIb in 38.2% (8/21) as per AGREE classification. Rates of delayed perforation were not significantly different between closed and open groups (4.7% vs 2.8%, *p* = 0.325). Univariate analysis revealed malignant polyps, procedure time and polyp size to be significantly associated with increased rate of delayed perforation (Table 6). Multivariate regression analysis revealed only malignant polyps (aOR:3.3, *p* = 0.02) were associated with increased risk of delayed perforation. Subgroup analysis in patients with malignant polyps, the rates of delayed perforation did not differ between closed and open groups(12.2% vs 3.8%, *p* = 0.39).

**Table 6:** Univariate and multivariate analysis for predictors of delayed perforations

	Delayed perforation (*n* = 21)		*p*-value
Univariate analysis			
Antiplatelet/anticoagulant use, Yes vs No, n (%)	4 (6.7%)	17 (3.4%)	0.269
Prior intervention, Yes vs No, n (%)	6 (5.7%)	15 (3.3%)	0.258
Right sided polyps, Yes vs No, n (%)	10 (3.7%)	11 (3.8%)	0.944
Paris 1 s or 1sp, Yes vs No, n (%)	10 (3.4%)	11 (4.5%)	0.543
Paris 2b, Yes vs No, n (%)	4 (4.3%)	17 (3.9%)	0.772
Traction, Yes vs No, n (%)	4 (2.7%)	17 (4.2%)	0.615
Submucosal fibrosis, Yes vs No, n (%)	14 (4.9%)	7 (2.6%)	0.165
Incomplete vs Complete resection, n (%)	0	21 (4%)	1.000
Prophylactic hemostasis, Yes vs No, n(%)	15 (4.7%)	6 (2.6%)	0.261
Closed vs Open defects, n (%)	17 (4.7%)	4 (2.8%)	0.325
Topical hemostatic agent, Yes vs No, n (%)	3 (4.4%)	18 (3.7%)	0.734
HGD, Yes vs No, n (%)	7 (5%)	14 (3.4%)	0.386
Malignant polyp, Yes vs No, n (%)	6 (8.1%)	15 (3.1%)	**0.048**
Mean (s.d.) age in years (perf vs no perf)	62.8 (11.4)	64.1 (11.6)	0.599
Mean (s.d.) procedure time in min (perf v no perf)	232.8 (202.3)	153.3 (111.1)	**0.002**
Mean (s.d.) polyp size in mm (perf. vs no perf)	55.2 (31.6)	42.7 (19.6)	**0.005**
Multivariate analysis			
Malignant polyps	3.3 (1.2–9.1)		0.02

*n* number, *s.d.* standard deviation, *HGD* high grade dysplasia

The* p*-value 0.05 and odds ratios with 95% confidence intervals not crossing 1 were considered as measures of statistical significance

### Overnight Hospital Admission

Overall 225 patients (44.5%) were admitted after ESD, and 281 patients (55.5%) were discharged the same day. Defect closure was associated with a reduction in overnight hospitalization compared to open defects with borderline statistical significance (41.8% vs 51.4%, *p* = 0.050). Table 7 shows regression analysis for predictors of post-ESD hospital admission. On this multivariate analysis, malignant polyps (*p* < 0.01), less traction use (*p* = 0.03), open ESD defects (*p* < 0.01) and less topical hemostatic agent use (*p* < 0.01) were associated with overnight hospital admission.

**Table 7:** Univariate and multivariate analysis for predictors of post-ESD hospital admission

	Hospital admission for observation (*n* = 257)		*p*-value
Univariate analysis			
Antiplatelet/anticoagulant use, Yes vs No, n (%)	31 (51.7%)	226 (45.3%)	0.349
Prior intervention, Yes vs No, n (%)	46 (43.4%)	211 (46.6%)	0.554
Right sided polyps, Yes vs No, n (%)	116 (43%)	141 (48.8%)	0.167
Paris 1 s or 1sp, Yes vs No, n (%)	158 (53.6%)	95 (38.6%)	** < 0.001**
Paris 2b, Yes vs No, n (%)	58 (63%)	195 (44.1%)	** < 0.001**
Traction, Yes vs No, n (%)	55 (36.9%)	202 (49.3%)	**0.010**
Submucosal fibrosis, Yes vs No, n (%)	137 (47.1%)	120 (44.8%)	0.585
Incomplete vs Complete resection, n (%)	9 (42.9%)	247 (46.1%)	0.771
Prophylactic hemostasis, Yes vs No, n(%)	157 (48.5%)	99 (42.3%)	0.150
Closed vs Open defects, n (%)	152 (41.8%)	73 (51.4%)	**0.050**
Topical hemostatic agent, Yes vs No, n (%)	25 (36.8%)	232 (47.3%)	0.104
HGD, Yes vs No, n (%)	63 (44.7%)	194 (46.4%)	0.721
Malignant polyp, Yes vs No, n (%)	46 (62.2%)	211 (43.5%)	**0.003**
Mean (s.d.) age in years (admission vs no admission)	64.5 (11.8)	63.7 (11.3)	0.444
Mean (s.d.) procedure time in min	153.1 (113.4)	160.6 (120.9)	0.465
Mean (s.d.) polyp size in mm	44.7 (21.9)	41.5 (18.5)	0.058
Multivariate analysis			
Malignant polyps	2.09 (1.23–3.55)		** < 0.01**
Traction use	0.645 (0.430–0.967)		**0.03**
Complete closure	0.560 (0.38–0.83)		** < 0.01**
Topical Hemostatic agent	0.422 (0.23–0.762)		** < 0.01**

*n* number, *s.d.* standard deviation, *HGD* high grade dysplasia, *DAE* delayed adverse events, *ESD* endoscopic submucosal dissection

The* p*-value 0.05 and odds ratios with 95% confidence intervals not crossing 1 were considered as measures of statistical significance

In subgroup analysis, of the 281 patients not admitted for overnight observation, prophylactic defect closure significantly reduced the rate of delayed bleeding (1% vs 5.8%, *p* = 0.036), and rate of all DAE (1.9% vs 7.2%, *p* = 0.043). Of the 225 patients who were admitted, defect closure was not associated with significant reduction of delayed bleeding, delayed perforation or overall DAE (Table 8).

**Table 8:** Subgroup analysis for defect closure and delayed adverse events (DAE) based on post-ESD overnight admission

Discharge vs Admission	Closed defects	Open defects	p-value
Overnight Admission (*n* = 225)			
Delayed bleeding	4 (2.6%)	4 (5.5%)	0.278
Delayed perforation	16 (10.5%)	4 (5.5%)	0.213
All DAE	29 (19.1%)	11 (15.1%)	0.461
Same day Discharge (*n* = 281)			
Delayed bleeding	2 (1%)	4 (5.8%)	**0.036**
Delayed perforation	1 (0.5%)	0	1.000
All DAE	4 (1.9%)	5 (7.2%)	**0.043**

The* p*-value 0.05 and odds ratios with 95% confidence intervals not crossing 1 were considered as measures of statistical significance

### Effect of Closure Types on Delayed Adverse Events (DAE)

**Fig. 3:**
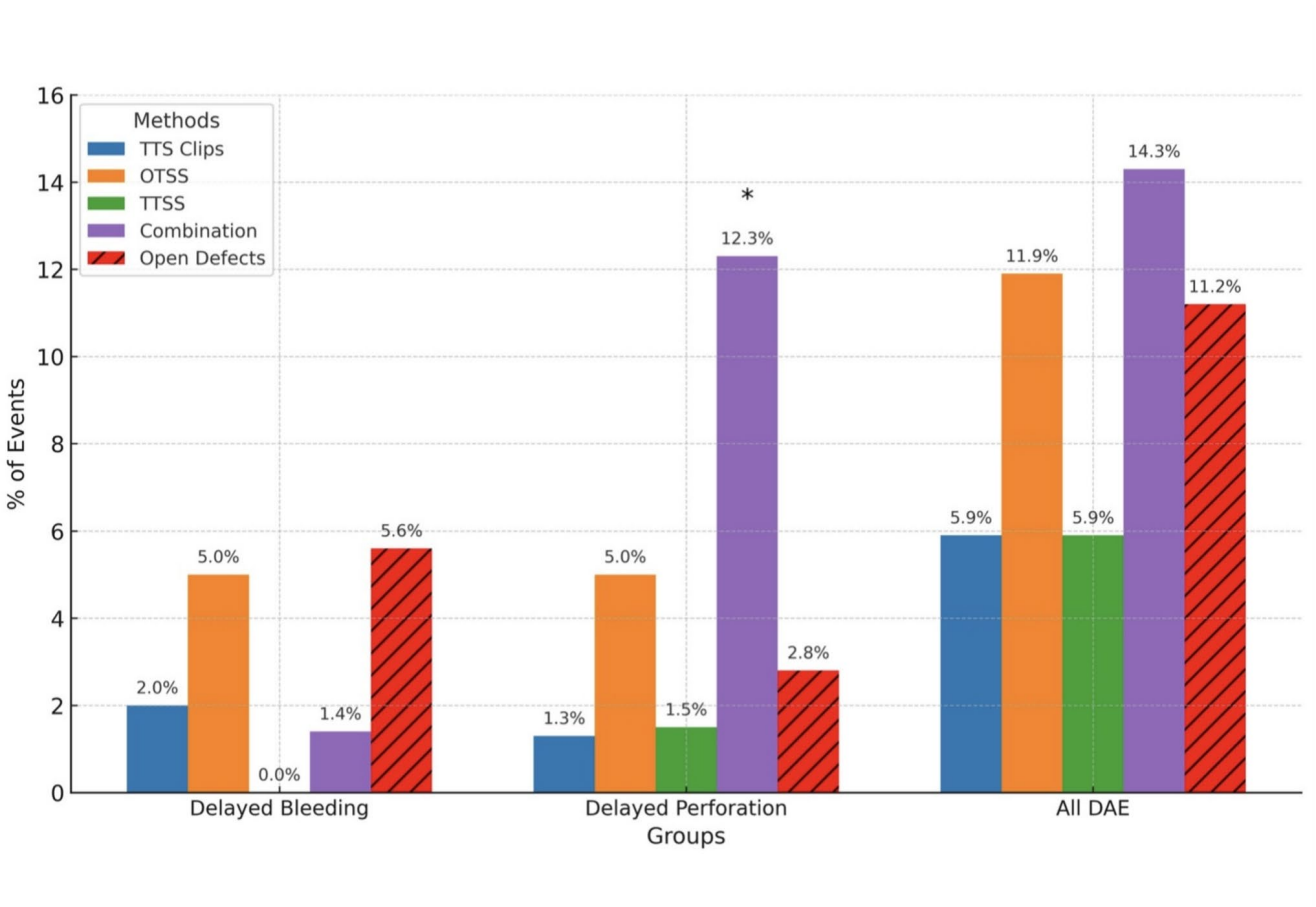
Demonstration of the rates of delayed bleeding, delayed perforation and all delayed adverse events based on closure type compared to the open group. *demonstrates statistically significant

Baseline characteristics of lesions included in each type of defect closure cohort has been presented in supplementary Table 1. The mean polyp size in mm for TTS clip use was significantly smaller (35.4 ± 15.3 vs 43.9 ± 21.5; *p* < 0.001) compared to the rest of the study polyps. On the other hand, the mean polyp size in mm for OTSS use was significantly larger (53.4 ± 24.0 vs 43.9 ± 21.5; *p* = 0.016) compared to the rest of the study polyps. TTS clips followed by TTSS were the most common single modalities used for defect closure. Both of these closure techniques were associated with numerical reductions in delayed bleeding, delayed perforation and overall DAE. These differences, however, were not statistically significant. Combination technique for defect closure was associated with a significantly higher risk of delayed perforation (12.3% vs 2.8%, *p* = 0.012). Figure 3 and supplementary Table 2 outlines the rates of DAE based on closure type compared to the open group. Supplementary Fig. 1 demonstrates a visual abstract summarizing important study outcomes.

## Discussion

Our large international multicenter study demonstrates significant reduction in delayed bleeding but not delayed perforation with prophylactic defect closure after colon ESD. On average, around 25 patients needed their colon ESD defects closed prophylactically to prevent one episode of delayed bleeding within 2 weeks of the procedure (number needed to treat, NNT = 25.6). In right sided polyps which was one of the high risk groups identified on multivariate analysis for delayed bleeding, the benefit of defect closure was further amplified with an absolute risk reduction (ARR) of 8% and NNT of 12.5. Defect closure was also associated with a significant reduction in post-procedure hospital admission.

This is one of the largest Western studies (*n* = 560) focusing on defect closure and DAEs after colon ESD. A prospective multicenter open label randomized control trial (EPOC trial, *n* = 249) from Japan demonstrated 13.4% (20.1% vs 6.7%) ARR after prophylactic clipping of LNPCPs, with multivariate analysis proving it as protective factor (OR: 0.2) for delayed bleeding [17]. Overall delayed bleeding rates were lower (2.7%) at baseline in our study, likely due to exclusion of rectal lesions and grade 1 AE that did not result in any intervention, and differences in closure techniques and devices used in our study. TTS clips were utilized for defect closure in the EPOC trial with 90% success rate using clips. The mean size of the polyps in the EPOC trial was 30 mm. In our study, the polyps were larger with mean size over 40 mm which explains the lower utilization (43% in closed group) of TTS clips. TTS clips, in our study, were used to close defects with average polyp size around 35 mm, while larger polyps (mean 53 mm) were closed with OTSS. EPOC trial also demonstrated rectal location to be a significant risk factor (OR:7.48) for delayed bleeding after ESD of LNPCPs, which was examined in our previous study^10^ and hence was excluded from this study. While there were differences in study design and lesion characteristics, like the EPOC trial, our study also demonstrated that prophylactic defect closure after ESD reduced the risk of delayed bleeding. While there were no differences in location within the colon in EPOC trial, our study demonstrated a higher risk of delayed bleeding and a higher risk reduction (ARR from 4.9% in the overall colon to 8% for the right colon) with defect closure in the right colon. These differences may arise due to thinner wall structures and robust blood supply, making it susceptible to injury. Moreover, increased mobility in right colon can make colonoscope maneuvering difficult and defect closure challenging compared to left colon.

In another recently reported Japanese study, the ABCD-J working group demonstrated 5.7% ARR after defect closure of colorectal ESD in patients on anticoagulants [18]. The benefit of defect closure for prevention of delayed bleeding was observed in right sided lesions (absolute risk reduction, 6.7%, *P* = 0.04), but not for left sided lesions. While our study found the use of anticoagulant and antiplatelet agents as an independent risk factor (aOR = 6.6), prophylactic defect closure did not result in a significant reduction in delayed bleeding, suggesting that pharmacologic bleeding risk may not be fully compensated by mechanical closure. While both studies are retrospective in nature, there are a few notable differences. The ABCD-J study had a much higher rate of delayed bleeding in patients with anticoagulant/antiplatelet use (> 10%) compared to our study (6.7%). This could be the result of comparison across two different sets of populations with different body constitutions or different endoscopic techniques, but also notably, the Japanese guidelines on perioperative management of antiplatelets and anticoagulants are significantly different compared to the Western guidelines [19]. For example, the short-acting direct oral anticoagulants (DOACs) are stopped on the day of ESD in Japan while they are typically stopped 48–72 h before ESD in the United States. TTS Clip closure was the only closure method used in the Japanese study. In our study, several closure methods were used. TTS clips and TTSS closures had numerical reductions in delayed bleeding, but these differences were not statistically significant. Similar to the ABCD-J study, the benefit of defect closure was amplified in right colon polyps but this was independent of the use of anticoagulation.

Prophylactic application of topical hemostatic agents (THAs) has garnered attention for prevention of delayed bleeding [20]. In a recent study, prophylactic application of self-assembling peptide significantly reduced delayed bleeding (OR:0.516) after colorectal ESD [21]. We did not find any difference in rates of delayed bleeding after THAs application in patients with open defects after ESD, likely related to much smaller number of patients (*n* = 19) who underwent THA application in our study.

Data on outcomes of prophylactic closure on delayed perforations after colonic ESD is scant. Intra-procedural perforations resulting from mechanical trauma can often undergo endoscopic repair. However, delayed perforations occur from thermal injury from repetitive hemostatic and coagulative procedures during ESD resulting in tissue necrosis, rather than mechanical injury, and associated with re-hospitalizations and surgery [22, 23]. Defect closure was not associated with reduced risk of delayed perforation in our study (4.7% vs 2.8%, *p* = 0.325). Previously, a multicenter randomized controlled trial demonstrated similar results [12]. TTS clips and TTSS had numerical reductions in delayed perforations without achieving statistical significance. This is likely due to overall smaller number of delayed perforations in our study.

Combination closure techniques were associated with higher rates of delayed perforation and all DAE. While we do not have the information on the reason behind choosing type of closure modality, we speculate that combination techniques were used when primary closure technique did not achieve complete closure to the endoscopists satisfaction. Higher rates of delayed perforation in the “combination closure technique” cohort likely represents challenges during closure rather than efficacy of the technique. Based on our data, we do not have enough information to say one closure modality is better than the other and future studies comparing efficacy of different closure techniques for closure of post ESD defects should be performed.

Majority of ESD is performed as an outpatient procedure in the West [24]. Though post-procedural admission for observation is common in Asia, same day discharge is commonly practiced in the West [25]. Patients with comorbidities, depressed polyp morphology, invasive cancer on histopathology and longer procedure times can be admitted for post-procedural monitoring [25]. It has been suggested to admit high risk patients selectively for post-ESD observation to avoid strain on the hospital resources [25]. Our study showed that defect closure can lead to significantly reduced (aOR: 0.56) chances of hospital admission for post-operative observation. Endoscopists’ decision to admit post-procedurally balances the risk of adverse events, and social support with increased cost of hospitalization. Cost-effective analysis of prophylactic clipping after large colorectal lesion EMR was found to be beneficial in high risk patients [26]. Overall, cost of closure devices vary by the type used. Previously, we demonstrated cost-effectiveness of TTSS over OTSS for closure of defects < 35 mm [27]. In our subgroup analysis, defect closure significantly reduced delayed bleeding (ARR 4.8%, NNT = 20.8) and all DAE (ARR 5.3%, NNT = 18.9). The average cost of hospitalization in the United States is nearly $12,000. The estimated cost of through the scope endoclips is $150–200 [28, 29], while TTSS costs $695 and OTSS can cost upto $ 1200 [28]. Univariate analysis revealed a 10% reduction in overnight hospital admission after ESD. A cost-effective strategy would be the one in which the defect could be closed with estimated cost of closure being less than $1200. Defect closure is likely to be cost-neutral if the defect can be closed with 6–8 endoclips or 1–2 TTSS or 1 OTSS. If we factor in the additional cost of interventions that result from DAE, closure will likely be cost-effective similar to the colorectal EMR data^23^. Studies focusing on cost-effective analysis of prophylactic ESD defect closure are warranted. In our study, we show that prophylactic defect closure after colon ESD can facilitate same day discharge with a significant reduction in delayed bleeding, DAE and potentially reduce the need for re-admission related to these events.

Although this is one of the largest studies on outcomes of prophylactic colon ESD defect closure on post-ESD delayed adverse events, there are still several limitations. First, the retrospective nature of the study relies on the accuracy of recorded data, which can affect the reliability of findings. We ensured the data from each center was as accurate as possible. Since our definition of DAE needed intervention or unplanned hospitalization, AGREE grade 1 events were not captured. Patients with mild hematochezia or post-electrocautery coagulation syndrome who were not re-admitted for observation, transfusion, antibiotics or intervention may be under-represented, and potential benefit might have been under-estimated. This is different from some of the other recently published data in this area and caution needs to be exercised comparing across studies.

The indication of ESD and final lesion selection, the defect closure and the decision to admit overnight for observation was up to the discretion of the endoscopist and guided by local institutional protocols and resource availability, thus, at risk of selection bias. The decision to close the defect and admit for observation is typically individualized due to lack of standardized recommendations and data. Typically the defect closure is performed after ESD in the right colon, patients on anticoagulation or patients with lack of immediate healthcare access after discharge. We did not have data on prior tattoo placement, which could affect fibrosis, but the degree of fibrosis was accounted for in the analysis. Lack of standardization of lesion selection and criteria for hospitalization after ESD raises the possibility of bias which are inherent to multicenter retrospective study design. Decision to prophylactically coagulate visible vessels during submucosal dissection were up to the endoscopist performing the procedure, and hence, could not be standardized. We performed multivariate analysis at each outcome to balance the effect of confounders as best as possible. Due to multicenter nature of the study, there could be variability in practice patterns, endoscopists experience across centers and endoscopists. It's also likely that endoscopists technique and skillset would change over the study period, and different centers and endoscopists would have varying experience and preferences for closure techniques, which was not accounted for in the study. However, performing a multicenter study may show real-world practice patterns and may allow for generalizability of the findings. The strengths of the study besides the large sample size include exclusion of partially closed defects for outcome analysis, multivariate analyses to adjust for the effect of confounders and subgroup analyses to quantify the effect of defect closure in certain groups that are at a higher risk of an outcome measure.

In conclusion, prophylactic closure of colon ESD defects was associated with a significant reduction in delayed bleeding, and post-procedural overnight hospital admission, but not delayed perforation. The findings of this study suggest that, if feasible, ESD defects should be closed in the colon (NNT = 25.6 for delayed bleeding). High risk groups such as right sided colon polyps may have the most benefit (NNT = 12.5) in reduction of delayed bleeding with defect closure. Prophylactic defect closure also justifies same day discharge with reduction in delayed bleeding and all DAE in the discharged patients. A Western prospective randomized trial on prophylactic defect closure after colon ESD is needed to confirm these findings.

## Supplementary Information

Below is the link to the electronic supplementary material.

Supplementary file1 (DOCX 860 KB)

## Data Availability

No datasets were generated or analysed during the current study.

## Abbreviations

ESD: Endoscopic submucosal dissection
DAE: Delayed adverse events
LNPCP: Large non- pedunculated colon polyps
EMR: Endoscopic mucosal resection
TTS: Through-the-scope
DAT: Dual Action Tissue
HGD: High grade dysplasia
ARR: Absolute risk reduction
NNT: Numbers needed to treat
THA: Topical hemostatic agents
