# Supplementary material for: Effect of Prophylactic Colon ESD (Endoscopic Submucosal Dissection) Defect Closure on Post-ESD Outcomes: An International Multi-center Retrospective Study
Source: Dig Dis Sci. 2025 Nov 5;71(4):1480–92. doi: 10.1007/s10620-025-09518-6 (PMC13144240; doi:10.1007/s10620-025-09518-6)
Supplement: Supplementary file 1 — Supplementary file1 (DOCX 860 KB) [file 10620_2025_9518_MOESM1_ESM.docx]

**Supplementary table 1: Characteristics of patients who underwent defect closure with through the scope, over the scope suturing system, through the scope suturing system and combination**

|  | **TTS (n=201)** | **OTSS (n=42)** | **TTSS (n=68)** | **Combination (n=77)** |
| --- | --- | --- | --- | --- |
| Age in years, mean (s.d.) | 64.1 (11.7) | 60.9 (13.0) | 62.5 (11.1) | 64.6 (12.2) |
| Antiplatelet or anticoagulation use, n (%) | 17(8.5%) | 5 (11.6%) | 9 (13%) | 10(12.5%) |
| History of previous intervention*, n (%) | 30 (14.9%) | 8 (18.6%) | 9 (13%) | 28 (35%) |
| Right sided polyps, n (%) | 101 (50.2%) | 10 (23.3%) | 35 (50.7%) | 45 (56.3%) |
| Paris 0-Is or 0-Isp, n (%) | 106 (53.8%) | 29 (74.4%) | 44 (66.7%) | 42 (55.3%) |
| Paris 0-IIb, n (%) | 37 (18.8%) | 1 (2.7%) | 8 (12.9%) | 13 (17.3%) |
| Presence of Fibrosis, n (%) | 103 (51.2%) | 16 (37.2%) | 34 (49.3%) | 52 (65%) |
| Prophylactic hemostasis n (%) | 116 (58%) | 37 (86%) | 29 (42%) | 48 (60%) |
| Traction use, n (%) | 55 (27.4%) | 18 (41.9%) | 13 (18.8%) | 23 (28.7%) |
| Incomplete resection, n (%) | 6 (3%) | 0 (0%) | 0 (0%) | 3 (3.8%) |
| Polyp size in mm, mean (sd) | 37.3 (16.7) | 52.9 (23.9) | 46.6 (18.1) | 44.2 (17.4) |
| High grade dysplasia, n (%) | 43 (23.4%) | 19 (44.2%) | 29 (42%) | 22 (27.5%) |
| Malignant Polyps, n (%) | 21 (10.4%) | 4 (9.3%) | 9 (13%) | 9 (11.3%) |
| Procedure time in minutes, mean (sd) | 113.9 (72.9) | 310.1 (186.5) | 164.9 (91.2) | 165.4 (94.7) |

*history of endoscopic mucosal resection, snare polypectomy or surgery

Abbreviations: ESD: endoscopic submucosal dissection; n: number; sd: standard deviation; TTS: TTS: through the scope; OTSS: over the scope suturing system; TTSS: through the scope suturing system

**Supplementary table 2:** Delayed bleeding, perforation and all delayed adverse events based on defect closure type vs the open group

| **Delayed Bleeding** | **Closed defects** | **Open Defects (n=143)** | **p-value** |
| --- | --- | --- | --- |
| TTS Clips (n=201) | 3 (2%) | 8 (5.6%) | 0.099 |
| OTSS (n=42) | 2 (5%) |  | 1.000 |
| TTSS (n=68) | 0 |  | 0.056 |
| Combination (n=77) | 1 (1.4%) |  | 0.278 |
| **Delayed Perforation** |  | | |
| TTS Clips (n=201) | 2 (1.3%) | 4 (2.8%) | 0.434 |
| OTSS (n=42) | 2 (5%) |  | 0.616 |
| TTSS (n=68) | 1 (1.5%) |  | 1.000 |
| Combination (n=77) | 9 (12.3%) |  | **0.012** |
| **All DAE** |  | | |
| TTS Clips (n=201) | 9 (5.9%) | 16 (11.2%) | 0.101 |
| OTSS (n=42) | 5 (11.9%) |  | 1.000 |
| TTSS (n=68) | 4 (5.9%) |  | 0.219 |
| Combination (n=77) | 11 (14.3%) |  | 0.504 |

Abbreviations: TTS: through the scope; DAE: delayed adverse events; OTSS: over the scope suturing system; TTSS: through the scope suturing system

**Supplementary figure 1: A visual abstract summarizing study outcomes**

**
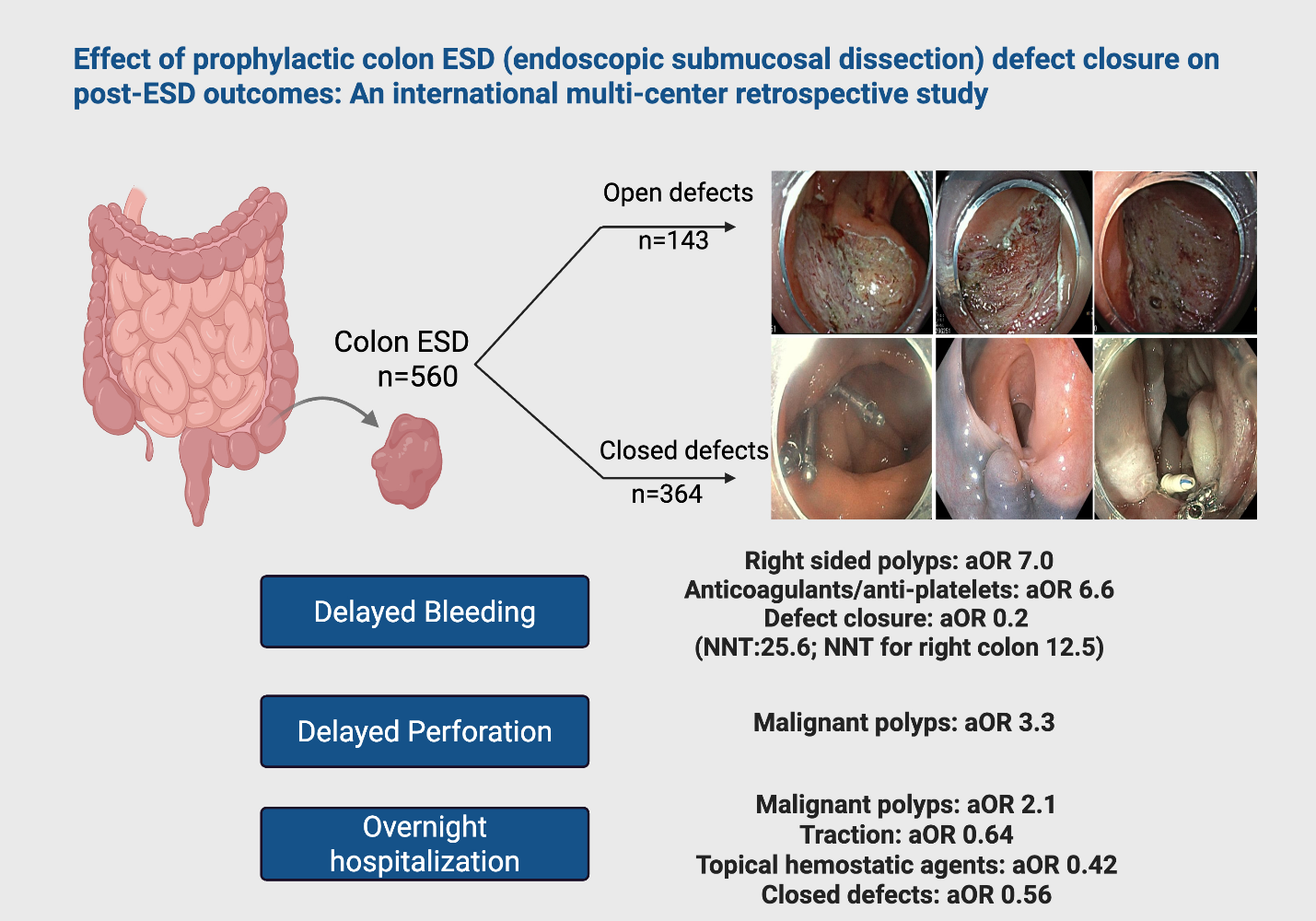
**
